# Short-term clinical effectiveness of 5% lidocaine patch after arthroscopic rotator cuff repair: study protocol for a randomized, double-blinded, placebo-controlled clinical trial

**DOI:** 10.1186/s13063-022-06886-6

**Published:** 2022-12-05

**Authors:** Yufan Qian, Yingjie Xu, Xiaohong Jin, Jiong Jiong Guo

**Affiliations:** 1 Department of Orthopedics and Sports Medicine, The First Affiliated Hospital of Soochow University, 188 Shizi Street, Suzhou, 215006 PR China; 2 Department of Traumatology, Dushu Lake Hospital Affiliated to Soochow University, Suzhou Industry Park, Suzhou, PR China; 3 Department of Anesthesiology, The First Affiliated Hospital of Soochow University, Suzhou, PR China

**Keywords:** Arthroscopic rotator cuff repair, Lidocaine patch, Randomized controlled trial, Pain management, Protocol

## Abstract

**Background:**

Arthroscopic rotator cuff repair (ARCR) often causes unbearable postoperative pain, even more severe than before surgery. Opioids are the drugs of choice for temporary postoperative analgesia. However, this conventional approach also has some side effects and potential for drug abuse. The aims of this study are expected to verify the effect of 5% lidocaine patch (LP5) on the intensity of early postoperative pain, functional recovery and quality of life in patients undergoing ARCR.

**Methods:**

In this randomized, double-blind, and placebo-controlled clinical trial, a total of 102 postoperative patients undergoing ARCR will be randomly assigned to either the LP5 group, receiving topical lidocaine analgesia, or the placebo control group. The primary outcome measure will be the change in the American Shoulder Elbow Surgeons score from pre-operation to 90 days post-operation. Secondary outcomes will include pain scores, range of motion, opioid use, safety indicators, blinding assessment and several shoulder function score questionnaires. The effect of the allocated treatment will be assessed at preoperative baseline and at 7-, 14-, 30- and 90-day postoperatively.

**Discussion:**

In this study, the efficacy and safety of the 5% lidocaine patch will be evaluated in terms of short-term clinical symptoms in patients undergoing ARCR. The results of this study will help determine whether LP5 is effective in early functional recovery in ARCR and whether it relieves pain and reduces opioid consumption.

**Trial registration:**

Chinese Clinical Trial Registry (http://www.chictr.org.cn) ChiCTR2200060108. Registered on 19 May 2022.

## Background

Arthroscopic rotator cuff repair (ARCR) has been proven to provide excellent clinical outcomes, such as restoration of tendon integrity and improvement in daily living, in patients with symptomatic rotator cuff tears who have failed to respond to conservative treatment [1, 2]. Although the short and long-term clinical outcomes of ARCR are very promising, postoperative pain is strongly associated with early return of mobility and patient satisfaction, particularly in intense postoperative pain [3]. Numerous methods of postoperative pain management after ARCR have been practised clinically, including oral and intravenous medications, regional nerve block, intralesional anaesthesia, periarticular injections [4, 5] and multimodal anaesthesia [6]. Opioids were once the cornerstone of post-ARCR pain management, however, their significant side effects and high addictive potential cannot be ignored as well [7–9]. Interscalene brachial plexus block (IBPB) also has some disadvantages, such as short timeliness, technical difficulty and a high probability of recurrence of pain [10, 11].

Five per cent lidocaine patch (LP5) is the first-line recommendation for postherpetic neuralgia and may reduce opioid consumption in acute postoperative pain [12–14]. Lidocaine is a voltage-gated sodium channel blocker. The proposed mechanism of action is to block abnormally expressed sodium channels following nerve injury, and lidocaine reduces abnormal peripheral nerve discharge and decreases peripheral sensitivity, thus exerting a local analgesic effect [15, 16]. In addition, the effectiveness of the topical lidocaine patch for other conditions, such as low back and myofascial pain, osteoarthritis of the knee and perioperative pain has been confirmed by previous studies [17–21].

To identify whether the lidocaine patch could produce similar beneficial effects in post-arthroscopic analgesia, we conducted a prospective double-blind randomized trial in patients undergoing ARCR. We hypothesized that lidocaine patch treatment immediately after ARCR would result in clinically and statistically significant improvements in pain levels, functional recovery and the use of oral analgesics.

## Methods

### Trial design

**Fig. 1:**
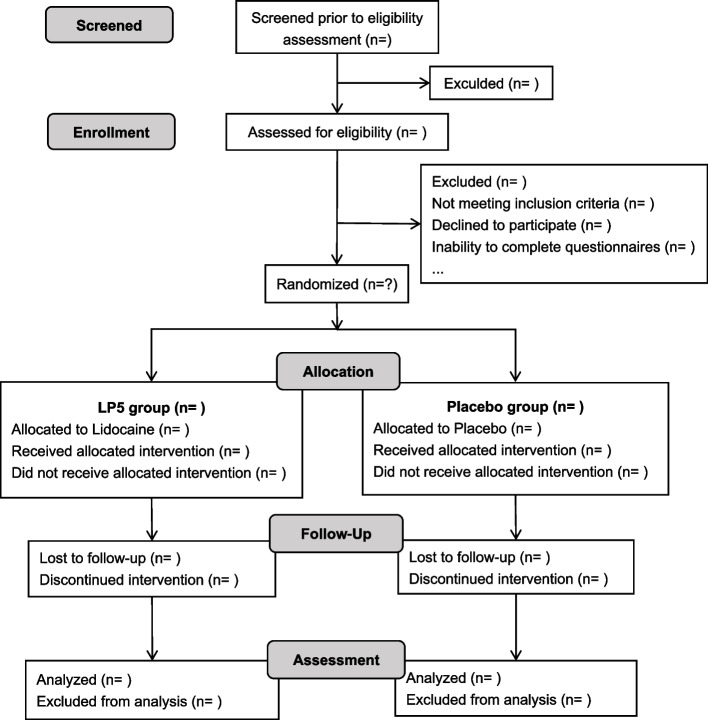
Study flow chart

The study will be a prospective, single-centre, randomized, placebo-controlled, double-blind, superiority trial in the First Affiliated Hospital of Soochow University conducted from May 2022 to December 2022, enrolling patients undergoing arthroscopic rotator cuff surgery and was registered in the Chinese Clinical Trial Registry (ChiCTR.org ID: ChiCTR2200060108). The study protocol has been approved by our institutional review board, and all subjects provided written informed consent. The flow chart of trial participation is given in Fig. 1.

### Recruitment and informed consent

This study will be conducted in the First Affiliated Hospital of Soochow University. Patients with rotator cuff tears requiring arthroscopic rotator cuff repair (ARCR) will be mobilized from our outpatient centre. After confirming the patient's eligibility for inclusion and completing a baseline level assessment, the investigator will give a detailed presentation of the study protocol and potential risks, etc., and answer all the questions raised by patients and their families. Each patient will sign an informed consent form. Upon enrolment, participants will be coded with a unique number.

### Eligibility

All patients confirmed with rotator cuff tear by MRI with symptoms (pain and/or weakness) and are failed to conservative treatments will be included. The exclusion criteria are (1) history of allergy to lidocaine or adhesive, (2) other significant organ disorders or substance abuse, (3) clear radiographic evidence of osteoarthritis of the glenohumeral joint, (4) presence of inflammatory arthritis including rheumatoid, (5) a history of ipsilateral shoulder dislocation or surgery, (6) irreparable tears, (7) pregnancy or heart disease, and (8) inability to understand questionnaires or express the level of pain.

### Withdrawal and termination criteria

Participants can withdraw from this trial in the following reasons:

1. Deciding to withdraw by themselves at any time and for any reason.
2. Loss to follow-up.
3. Experiencing serious adverse events or allergic reaction.

### Randomization and blinding

Prior to the start of the study, randomization will be performed using a computer-generated table of random numbers by the research pharmacy not involved in the screening of subjects. Included participants will be randomly assigned with a 1:1 allocation to either the lidocaine or placebo group, with each patient having an equal probability of being assigned to either the experimental treatment group or the placebo group. The material, size and colour of the placebo patch will be similar to LP5 as far as possible. Patients will use the patches under the guidance of their surgeons. The group allocation protocols will be enclosed in opaque envelopes to ensure blinding of the investigators, patients, and data analysts. These envelopes will be sealed and could only be opened after the study is completed.

If a subject experiences a serious adverse event or requires emergency resuscitation during the trial, whether or not it is related to the intervention, the intervention received by the subject will be disclosed. Subjects who disclose the intervention will be considered as drop-out cases. If the intervention is disclosed for more than 20% of participants, the trial will not be considered double-blind.

In addition, the allocation scheme of the intervention will be confidential to the evaluators collecting outcome data throughout the study. The statistical analysts will also not know whether participants are in the LP5 or placebo group until the statistical analysis is completed.

### Intervention

The group allocation protocol will be performed prior to the procedure. Based on the random number, a pre-prepared opaque envelope containing either 5% LP measuring 14 × 10 cm^2^ and containing 700 mg of lidocaine or placebo patches was allocated to the patient. There is no difference between the placebo patches and LP5 patch in appearance or structure, but the placebo does not contain lidocaine. The patch will be divided into strips and applied around the shoulder crest immediately after surgery. The patch will be replaced with a new one every 12 h until 2 weeks postoperatively. Patients, test site medical staff and investigators were blinded to group allocation. In cases of severe shoulder pain, painkillers will be allowed as emergency medication and should be documented in the medical record. Patients will also be asked to record the name, dose, date, frequency and exact time of medication used and to report to the researcher if they have taken any medication during the study. Patient compliance will be maintained and monitored through good communication and constant reminders between medical staff and patients. Although patients will be advised to adhere to the protocol, it is not possible to fully exclude those patients who independently decide to undergo and not report other treatments or measures. We will explain in detail to the patients and their family members simultaneously. A close follow-up and telemedicine will be performed during the trial.

### Surgical procedures and postoperative rehabilitation

All ARCR procedures will be performed with the patient in the lateral decubitus position under general anaesthesia. The limb to be operated on is attached to a skin traction device and 3 kg of weight is used to keep the shoulder in a position of 30°–60° of abduction and 20°–30° of flexion. After required arthroscopic portals have been established, an arthroscopic examination will be performed, the hyperplastic bursal tissue and footprint area will be cleared, and acromioplasty will be performed based on intraoperative assessment. The tear will be repaired as clinically indicated for the tear pattern using suture anchors. All patients will receive the same postoperative rehabilitation protocol with the assistance of physical therapists.

### Outcomes measurement

Participants will be scheduled for a 3-month follow-up, with data collected at baseline (pre-operative), 1-, 7-, 14-, 30- and 90-day postoperative using a uniform standardized case report form. Baseline clinical data will be collected including age, gender, dominant hand, affected limb, body mass index, comorbidities, body mass index, duration of symptoms, range of motion, previous treatment to the shoulder (including surgery), and details of all treatments within the last 12 months (including the use of intra-articular therapy) (Table 1).

**Table 1:** Schedule of study enrolment, interventions, and assessments

	Study period						
	Screening	Baseline	Treatment phase			Follow-up phase	
Timepoint	***-1 week***	***0***	***1-day***	***7-day***	***14-day***	***30-day***	***90-day***
**Enrolment**							
**Eligibility screen**	×						
**Informed consent**	×						
**Medical history**	×						
**Physical examination**	×						
**Comorbidity**							
**Allocation**		×					
**Interventions**							
**LP5 group**			×	×	×		
**Placebo group**			×	×	×		
**Outcomes**							
**ASES**		×				×	×
**OSS**		×				×	×
**CMS**		×				×	×
**ROM**		×				×	×
**SF-36**		×				×	×
**PSQI**		×	×	×	×	×	×
**VAS**		×	×	×	×	×	×
**PPT**		×	×	×	×	×	×
**Safety assessments**							
**BRE**		×					×
**URT**		×					×
**LFTs**		×					×
**ESR**		×					×
**ASO**		×					×
**RF**		×					×
**Opioid consumption**		×	×	×	×	×	×
**Adverse events**		×	×	×	×	×	×

*Abbreviations*: *LP5* 5% Lidocaine patch, *ASES* American Shoulder and Elbow Surgeons, *OSS* Oxford Shoulder Score, *CMS* Constant-Murley, *PSQI* Pittsburgh Sleep Quality Index, *SF-36* 36-Item Short Form Survey, *VAS* Visual analogue scale, *PPT* Pressure pain threshold, *BRE* Blood routine examination, *URT* Urine routine test, *LFTs* Liver function tests, *ESR* Erythrocyte sedimentation rate, *ASO* Anti-streptococcus hemolysinv, *RF* Rheumatoid factor

#### Primary outcome

The primary outcomes will be the changes from the baseline of the American Shoulder and Elbow Surgeons score (ASES) [22] at the completion of the 90-day follow-up.

#### Secondary outcome

##### Functional questionnaire assessment

Participants will complete a series of patient-reported questionnaires at the preoperative, 30-day and 90-day postoperative follow-up points to assess pain, function and patient satisfaction: Oxford Shoulder Score (OSS) [23], the Constant-Murley score (CMS) [24], Pittsburgh Sleep Quality Index (PSQI) [25] and the 36-Item Short Form Survey (SF-36) [26]. In addition to the change in ASES at the terminal follow-up, ASES at other secular nodes will also be included in the secondary outcomes. All measurements will be carried out by an independent blinded assessor.

##### Range of motion

With the patient standing, active and passive mobility of the affected and unaffected shoulder, including forward elevation (FE), external rotation (ER) and internal rotation (IR), will be recorded using a goniometer. For statistical convenience, active internal rotation of the back will be measured by recording the vertebral levels reached with the tip of the thumb. vertebral levels from T1 to T12 were numbered consecutively as 1 to 12; vertebral levels from L1 to L5 were numbered consecutively as 13 to 17 and any levels below the sacrum were numbered consecutively as 18 [27, 28].

##### Visual analogue scale

The visual analogue scale [VAS, scale from 0 (no pain) to 10 points (unbearable pain)] will be used to quantify perioperative pain at rest (VASr), pain at night (VASn) and during active movement (VASm).

##### Mechanical sensitivity

The Mechanical sensitivity will be measured with pressure pain threshold (PPT), defined as the amount of pressure applied for the pressure sensation to first transform to pain. PPT will be assessed using a 10-kgf analogic pressure algometer placed in the footprint area of the rotator cuff (Wagner Instruments, Greenwich, CT, USA). The pressure is slowly increased and the minimum value that allows the subject to report a shift to pain or discomfort is recorded. The average of three repeated measurements, with 30–60s rest intervals, will be calculated for statistical analysis [29, 30].

### Plans for collection and use of participant data and biological specimens in this trial/future use

Not applicable, as the study will not use participant data including biological specimens in the future.

### Statistical methods

#### Sample size calculation

There are no reports in the available literature relating to the primary outcome. A pre-trial with a sample size of 60 was conducted in our orthopaedic clinic. 3-month follow-up time results showed ASES scores of 63.56 ± 15.56 and 74.94 ± 18.42 in the control and intervention groups, respectively. With a type I error rate of 0.05 (*α* = 0.05, two-tail) and a power of 90% (*β* = 0.10), the sample size for the study protocol was calculated as 41 patients per group based on the primary outcome indicator by PASS 15.0 (Power Analysis and Sample Size, NCSS, LLC, USA). Assuming a 20% drop-out rate, we plan to recruit 102 participants (51 per group) to the study.

### Statistical analysis

The final data will be entered and counted by a professional statistician using SPSS 26.0 (IBM, Armonk, NY, USA). For continuous variables, the normality of measured data distributions will be evaluated using the Shapiro-Wilk test. Measurements will be expressed as mean ± standard deviation, if the data are normally distributed with uniform variance. Paired *t*-tests will be used for within-group comparisons before and after treatment, and independent samples *t*-tests will be used for between-group comparisons before and after treatment. If the data do not conform to a normal distribution or have an uneven variance, the measurement data will be expressed as median (interquartile range) [M(Q)] and the Wilcoxon rank sum test will be used for the analysis of the data before and after treatment. For categorical variables, chi-square tests or Fisher’s exact tests will be used to examine differences between groups and to describe effect sizes in terms of percentages and frequencies. Prior to any analysis, the pattern and reasons for missing data patterns will be investigated. The primary analysis will be conducted as an intention-to-treat analysis, which includes all participants with missing outcome data, unless there is clear evidence that its underlying assumption is inappropriate. Multivariable imputation by chained equations method will be used to impute missing data for the sensitivity analysis for the primary and secondary outcomes. *P*<0.05 indicates that the threshold of statistical significance has been reached.

### Data collection and management

After meeting all inclusion criteria and agreeing to participate, participants will be coded with a study number and all data referring to the patient will be recorded by this number rather than by name. Patients undergo routine preoperative laboratory and imaging assessments, including a review of preoperative medication and documentation of preoperative opioid use. A Case Report Form (CRF) will be used to collect information from participants, including baseline information, imaging results and follow-up visits. Shoulder mobility, clinical and demographic data, pain scores, and shoulder mobility scores will be collected at follow-up points. Data will also be collected on complications and adverse reactions, including death, reinfection, reoperation, medical complications, rash or allergic reactions. Independent researchers will manually enter the raw data storage. A Data Security Monitoring Board (DSMB) has been set up to review the reliability and security of the data. CRF as well as uploaded data will be stored in a secure cabinet or password-protected folder respectively. Access to study data by other researchers will be restricted. No interim analysis is planned for topical application of 5% lidocaine as the safety concerns are minimal unless required by the Steering Committee or DSMB because of safety concerns.

Acute pain after ARCR is a concern for patients. In addition, a close follow-up and telemedicine will be performed to reduce the rate of loss to follow-up. All patients will be followed up for 3 months for postoperative recovery and monitoring of complications. Patients will receive a text message or email two days before each postoperative follow-up point.

### Oversight and monitoring

For this single-centre trial, the trial staff has trained investigators who are responsible for assessing the subgroups and clinical data collection. Providing oversight and guidance for this trial is the Quality Management Office of the First Affiliated Hospital of Soochow University, who will review the data and check the progress of the study.

### Quality control

Prior to the trial, all staff will be required to attend a series of training sessions. These sessions will ensure that relevant staff fully understands the study protocol and the standard operating procedures of the study. In order to maintain the consistently high quality of clinical trials, the Clinical Research Centre of the First Hospital of Soochow University will regularly monitor study files, informed consent forms, case report forms (CRFs), serious AEs and data records.

### Adverse event management

Any adverse events (AEs) occurring to participants during the clinical trial, whether or not they are related to the intervention, will be assessed and recorded at any time. Adverse events will be recorded on a designated Clinical Case Observation Form (CRF) Adverse Event Form and classified according to the Medical Dictionary of Regulatory Activities (MedDRA). During this period, if the participant feels a slight burning, tingling, redness, blistering or breakage of the local skin, the area will be disinfected and covered with sterile gauze to prevent infection. Other side effects caused by lidocaine such as: hypotension, dizziness, slowed heart rate and gastrointestinal reactions will be recorded on the CRF. Drug safety will be assessed by blood routine examination (BRE), urine routine test (URT), liver function tests (LFTs) and other indicators of significant organ damage. Indicators of joint cavity infection such as erythrocyte sedimentation rate (ESR), anti-streptococcus hemolysinv (ASO) and rheumatoid factor (RF) will be recorded. Data will be collected on complications and adverse reactions, including death, reinfection, reoperation, medical complications, rash or allergic reactions. Serious adverse events (SAEs) will have to be reported to the Ethics Committee of the First Affiliated Hospital of Soochow University within 24 h. In the event of a medical emergency, random coding and group allocation of individuals can be determined through standard operating procedures. These complications will be included in the secondary outcome of this study.

### Plans for communicating important protocol amendments to relevant parties (e.g. trial participants, ethical committees)

If modifications to the protocol are necessary, these will be submitted to our hospital ethics committee and the Chinese Clinical Trial Registry. Following approval by the relevant units, study participants will be informed that they may withdraw their consent at any time.

## Discussion

This paper presents a double-blind, placebo, randomized controlled trial designed to investigate the efficacy of LP5 for short-term analgesia after ARCR and whether it reduces the consumption of oral opioids. The analgesic effect of the LP5 is mainly due to the blocking of afferent nociceptive transmission. Locally released lidocaine is absorbed by the pain fibres of the skin, blocking sodium channels in the neuronal membrane and preventing the transmission of action potentials from the periphery to the cerebral cortex. In addition, lidocaine inhibits the activation of neutrophils and reduces the release of cytokines, thereby reducing the acute phase of the inflammatory response [31, 32]. There was evidence in the literature to support the view that lidocaine patches were effective in reducing postoperative pain and opioid consumption for laparoscopic gynaecological surgery, appendectomy and radical prostatectomy [12, 20, 33]. However, there is a lack of valid evidence for the effectiveness of ARCR postoperatively. It should be noted that this intervention is low cost and can be conveniently implemented in the postoperative period. One of the limitations of this trial is the small sample size of this trial. Therefore, a multicentre trial with a large sample size is necessary.

## Trial status

The trial is currently in the recruitment phase. The study will run from May 2022 to December 2022.

## Data Availability

Trial data are available on reasonable request to the principal investigator of the study.

## Abbreviations

ARCR: Arthroscopic rotator cuff repair
LP5: 5% lidocaine patch
IBPB: Interscalene brachial plexus block
ASES: American Shoulder and Elbow Surgeons
OSS: Oxford Shoulder Score
CMS: Constant-Murley
PSQI: Pittsburgh Sleep Quality Index
SF-36: 36-Item Short Form Survey
FE: Forward elevation
ER: External rotation
IR: Internal rotation
VAS: Visual analogue scale
PPT: Pressure pain threshold
AEs: Adverse events
BRE: Blood routine examination
URT: Urine routine test
LFTs: Liver function tests
ESR: Erythrocyte sedimentation rate
ASO: Anti-streptococcus hemolysin
RF: Rheumatoid factor
SD: Standard deviation
